# Abundant resistome determinants in rhizosphere soil of the wild plant *Abutilon fruticosum*

**DOI:** 10.1186/s13568-023-01597-w

**Published:** 2023-08-30

**Authors:** Wafa A. Alshehri, Aala A. Abulfaraj, Mashael D. Alqahtani, Maryam M. Alomran, Nahaa M. Alotaibi, Khairiah Alwutayd, Abeer S. Aloufi, Fatimah M. Alshehrei, Khulood F. Alabbosh, Sahar A. Alshareef, Ruba A. Ashy, Mohammed Y. Refai, Rewaa S. Jalal

**Affiliations:** 1 Department of Biology, College of Science, University of Jeddah, 21493 Jeddah, Saudi Arabia; 2 Biological Sciences Department, College of Science & Arts, King Abdulaziz University, 21911 Rabigh, Saudi Arabia; 3 Department of Biology, College of Science, Princess Nourah bint Abdulrahman University, P.O.Box 84428, 11671 Riyadh, Saudi Arabia; 4 Department of Biology, Jumum College University, Umm Al-Qura University, P.O. Box 7388, 21955 Makkah, Saudi Arabia; 5 Department of Biology, College of Science, University of Hail, Hail, Saudi Arabia; 6 Department of Biology, College of Science and Arts at Khulis, University of Jeddah, 21921 Jeddah, Saudi Arabia; 7 Department of Biochemistry, College of Science, University of Jeddah, 21493 Jeddah, Saudi Arabia

**Keywords:** ARG, HGT, mWGS, MGEs, AMR, Prevalence, Rhizosphere, Microbiome, Antibiotic resistance mechanism

## Abstract

A metagenomic whole genome shotgun sequencing approach was used for rhizospheric soil micribiome of the wild plant *Abutilon fruticosum* in order to detect antibiotic resistance genes (ARGs) along with their antibiotic resistance mechanisms and to detect potential risk of these ARGs to human health upon transfer to clinical isolates. The study emphasized the potential risk to human health of such human pathogenic or commensal bacteria, being transferred via food chain or horizontally transferred to human clinical isolates. The top highly abundant rhizospheric soil non-redundant ARGs that are prevalent in bacterial human pathogens or colonizers (commensal) included *mtrA*, *soxR, vanRO*, *golS*, *rbpA*, *kdpE*, *rpoB2*, *arr-1*, *efrA* and *ileS* genes. Human pathogenic/colonizer bacteria existing in this soil rhizosphere included members of genera *Mycobacterium*, *Vibrio*, *Klebsiella*, *Stenotrophomonas*, *Pseudomonas*, *Nocardia*, *Salmonella*, *Escherichia*, *Citrobacter*, *Serratia*, *Shigella*, *Cronobacter* and *Bifidobacterium*. These bacteria belong to phyla *Actinobacteria* and *Proteobacteria*. The most highly abundant resistance mechanisms included antibiotic efflux pump, antibiotic target alteration, antibiotic target protection and antibiotic inactivation. antimicrobial resistance (AMR) families of the resistance mechanism of antibiotic efflux pump included resistance-nodulation-cell division (RND) antibiotic efflux pump (for *mtrA*, *soxR* and *golS* genes), major facilitator superfamily (MFS) antibiotic efflux pump (for *soxR* gene), the two-component regulatory kdpDE system (for *kdpE* gene) and ATP-binding cassette (ABC) antibiotic efflux pump (for *efrA* gene). AMR families of the resistance mechanism of antibiotic target alteration included glycopeptide resistance gene cluster (for *vanRO* gene), rifamycin-resistant beta-subunit of RNA polymerase (for *rpoB2* gene) and antibiotic-resistant isoleucyl-tRNA synthetase (for *ileS* gene). AMR families of the resistance mechanism of antibiotic target protection included bacterial RNA polymerase-binding protein (for *RbpA* gene), while those of the resistance mechanism of antibiotic inactivation included rifampin ADP-ribosyltransferase (for *arr-1* gene). Better agricultural and food transport practices are required especially for edible plant parts or those used in folkloric medicine.

**Supplementary Information:**

The online version contains supplementary material available at 10.1186/s13568-023-01597-w.

## Key points

- Risk of transfer of rhizosphere resistome of *A. fruticosum* to human gut was explored
- The most common resistance mechanism in this resistome is antibiotic efflux pump
- Rhizobiome is a source of new antibiotics with feasible therapeutic interventions

**Supplementary Information:**

The online version contains supplementary material available at 10.1186/s13568-023-01597-w.

## Introduction

*Abutilon fruticosum* is a perennial medicinal plant species of family *Malvaceae* that is native to northern America, Africa and southwestern and western Asia including Saudi Arabia (Alzahrani 2021; Fryxell 2002). Species of the genus *Abutilon* have high medicinal and economic benefits as some plant parts, such as leaf, root and seed, have important therapeutic merits (Patel and Rajput 2013). For example, plant leaves were proven to contain useful steroids, carbohydrates and flavonoids, while root can contain linoleic acid, oleic acid, and palmitic acid, and seed embraces the essential amino acids (Suryawanshi and Umate 2020). As members of this genus are not toxic, they can be eaten orally by humans to relief body pain (Khadabadi and Bhajipale 2010; Suryawanshi and Umate 2020). They can also be used in the treatment of ulcers, piles, inflammation of the bladder, etc. (Husain and Baquar 1974; Patel and Rajput 2013).

Metagenomic whole genome shotgun sequencing (mWGS) is an approach to detect accurate signature of soil microbiome down to the strain level (Bai et al. 2015). The capability to explore not only the archaeome and bacteriome but also fungi and virome is another advantage of the mWGS (or gene cataloguing) method. This approach also provides accurate insights into evolution, assembly, shaping, and diversity of plant rhizospheric microbiomes (Bai et al. 2015; Segata et al. 2011; Vorholt 2012; Vorholt et al. 2017). Furthermore, mWGS can accurately assign microbial function and contribution to the intact environment and technically allows the study of soil resistome, CAZymes (Carbohydrate Active enZymes) and KEGG (Kyoto Encyclopedia of Genes and Genomes) pathways.

Rhizospheric region is regarded as the host or theatre of plant–microbe interactions and as a machinery for the recovery of new bacterial genes and strains. Until recently, no enough attention was paid to the possible occurrence of horizontal transfer of antibiotic resistance genes (ARGs) existing in rhizospheric microbiomes, especially those of native or wild plant species (Obermeier et al. 2021; Peterson and Kaur 2018). It is anticipated that rhizobiome of wild plants allows the detection of new antibiotics in its antibiotic producer microbes, like members of genus *Streptomyces*, along with new versatile ARGs. The latter events occur due to the highly diversified microbial communities being exposed to selective pressure in the wild habitat and to the highly diversified secondary metabolic processes harbored by bacteria (Berendonk et al. 2015). Such new genotypes might acquire the potentiality to horizontally transfer ARGs existing in their mobile genetic elements (MGEs), like plasmids, phages and integrons, to genetically-related human pathogens in gut microbiome (Bäckhed et al. 2015; Blau et al. 2019; Chen et al. 2019). Risk of horizontal gene transfer (HGT) is high when human pathogenic bacteria harboring ARGs exist in the rhizospheric soil region (Nelkner et al. 2019). The risk becomes higher when rhizosphere is for an edible plant by human or livestock or a plant with medicinal and/or commercial values, like *A. fruticosum*, thus is a subject to enormous human activities (Raes et al. 2007; Tringe et al. 2005). Even human commensal microbes, like *Pseudomonas aeruginosa*, can approach virulence and become opportunistic pathogens when they acquire new ARGs and become antibiotic self-resistant (Miller et al. 2014). Other examples of such bacterial “superbugs” include *Mycobacterium tuberculosis*, *Staphylococcus aureus* and *Acinetobacter baumannii* (Peterson and Kaur 2018). The prior well-documented example refers to mobilization and transfer of the chromosomal β-lactamase gene *amp*C to gram-negative clinical isolates (Aryal et al. 2020).

The present study aimed to detect resistome signature of soil-dwelling rhizospheric bacteria of the wild plant species *Abutilon fruticosum* along with the accompanied antibacterial resistance mechanisms. More focus was given to abundant soil bacteria that were well-documented to be pathogenic or colonizers to human. The study also aimed to investigate the possible risk of horizontal transfer of antibiotic resistance genes (ARGs) to pathogenic bacteria in human gut.

## Materials and methods

### Sample collection and DNA extraction

Microbial samples were collected in three replicates from rhizospheric soil of *Abutilon fruticosum* plants growing naturally in the North Western region of Mecca district, Saudi Arabia (Al-Eisawi and Al-Ruzayza 2015). Region selected for the experiment received no rainfall for > 3 months prior collection, while selected plants are single-grown and have homogenous performance. Concurrently, three bulk soil samples were taken ~ 10 m apart from the three selected plants. Collected soil samples were immediately put in liquid nitrogen and stored at − 20 °C until use (Hurt et al. 2001). Metagenomic DNAs of different soil samples were extracted using CTAB/SDS method and DNA concentration was adjusted to 10 ng/μl as described (Tashkandi et al. 2022). DNA extraction buffer consisted of 10 ml Tris-HCl (1 M), 4 ml M EDTA (0.5), 2 g CTAB and 28 ml NaCl (5 M). After centrifugation at 12,000 xg, precipitated DNA was washed in 0.5 ml chilled ethanol (70%) and finally suspended in 1 ml TE buffer. RNA residuals were removed by adding 10 ul RNase A and incubate DNA solution at 37 °C for 1 h.

### Whole genome shotgun sequencing and bioinformatics analysis

DNA samples (30 ul each) were shipped to Novogene Co. (Singapore) for whole metagenome shotgun sequencing. Based on the quality control criterion, reads with low quality bases of ≥ 40 bp and with N nucleotides of > 10 bp were removed. Then, library preparation was done and clean data were sequenced on Illumina HiSeq 2500 platform as described (Tashkandi et al. 2022). Generated data were assembled using MEGAHIT (K-mer = 55) and chimeras were removed as described (Karlsson et al. 2012; Mende et al. 2012; Oh et al. 2014). NOVO_MIX scaffolds were generated from unassembled less-abundant reads of different samples. Recovered scaftigs were mapped and non-redundant genes were checked and predicted for function via MetaGeneMark (Mende et al. 2012; Nielsen et al. 2014) and dereplicated using Cluster Database at High Identity with Tolerance (CD-HIT) (Fu et al. 2012; Li and Godzik 2006). Then, gene catalogues (nrGC) were constructed using a greedy pairwise comparison (Li et al. 2014) and annotated using the binning reference-based classification method MEGAN (Huson et al. 2011; 2016).

Proteins encoded by non-redundant genes were mapped against CARD (https://card.mcmaster.ca/ontology/) (e value ≤ 1^e−5^) (Martínez et al. 2015) and antibiotic resistance genes (ARGs) were recovered and checked for abundance (Forsberg et al. 2014; Yang et al. 2013), then, categorized to antimicrobial resistance (AMR) families and antibiotic resistance mechanisms as described (Liu and Pop 2009). Then, a circle chart was drawn to show the overall proportion and distribution of the most abundant ARGs against samples of the two soil types. The chart is divided into two parts where the first refers to soil types (right side) and the second refers to the most abundant ARGs (left side). Colors in the right side in inner circle refer to sum of relative abundance of all ARGs per sample, while, color in the left side refer to sum of relative abundance of all samples per ARG. Colors in the right side of outer circle refer to detailed relative abundance of different ARGs per sample, while, colors in the left side refer to detailed relative abundance of different samples per ARG.

## Results

Blastp with CARD (the Comprehensive Antibiotic Research Database) was used to detect unique ORFs referring to the antibiotic resistance genes (ARGs) in rhizosphere (R) and bulk (S) soil microbiomes of *Abutilon fruticosum*. Alignment results indicate the occurrence of 1837 gene queries generated either from assembled reads of one soil sample (R or S) (812 gene queries) or from re-assembled less abundant reads of all samples (NOVO_MIX) (1025 gene queries) (Additional file 1: Table S1). Selected identity percentage of gene query sequences with those in the NCBI (National Center for Biotechnology Information) subjects is ≥ 50, while allowed percentage of mismatch is ≤ 50%. The results in Table S2 indicate assignment of 1296 gene queries to specific ARGs of which 590 refer to one soil sample, while 708 refer to NOVO_MIX reads (Additional file 2: Table S2).

**Fig. 1:**
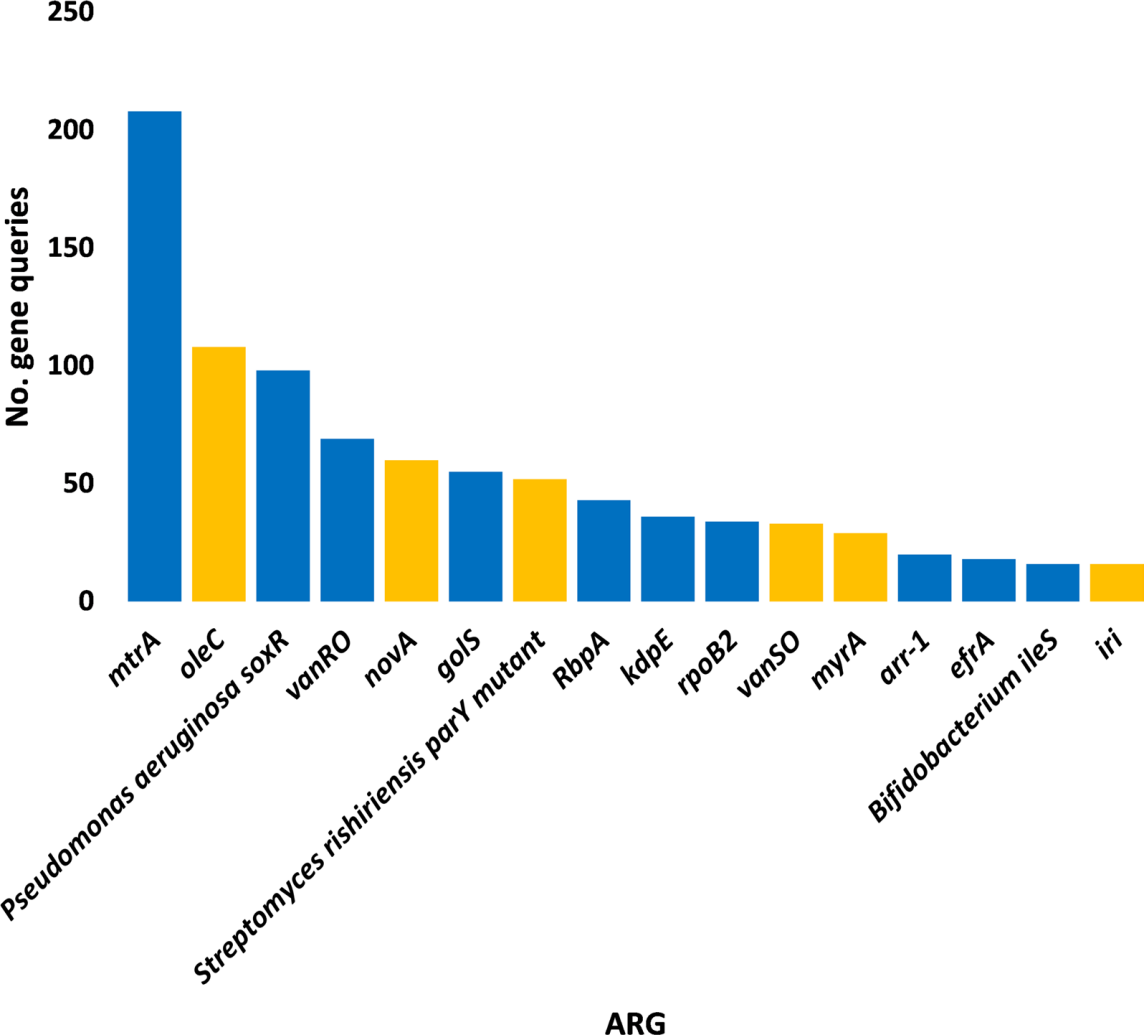
Query number of the most abundant (> 15) non-redundant antibiotic resistance genes (ARGs) generated from soil microbiomes of *Abutilon fruticosum*. Orange columns refer to ARGs that are not prevalent in human pathogens or bacterial colonizers. See Additional files 3,4: Tables S3 and S4 for more details

**Fig. 2:**
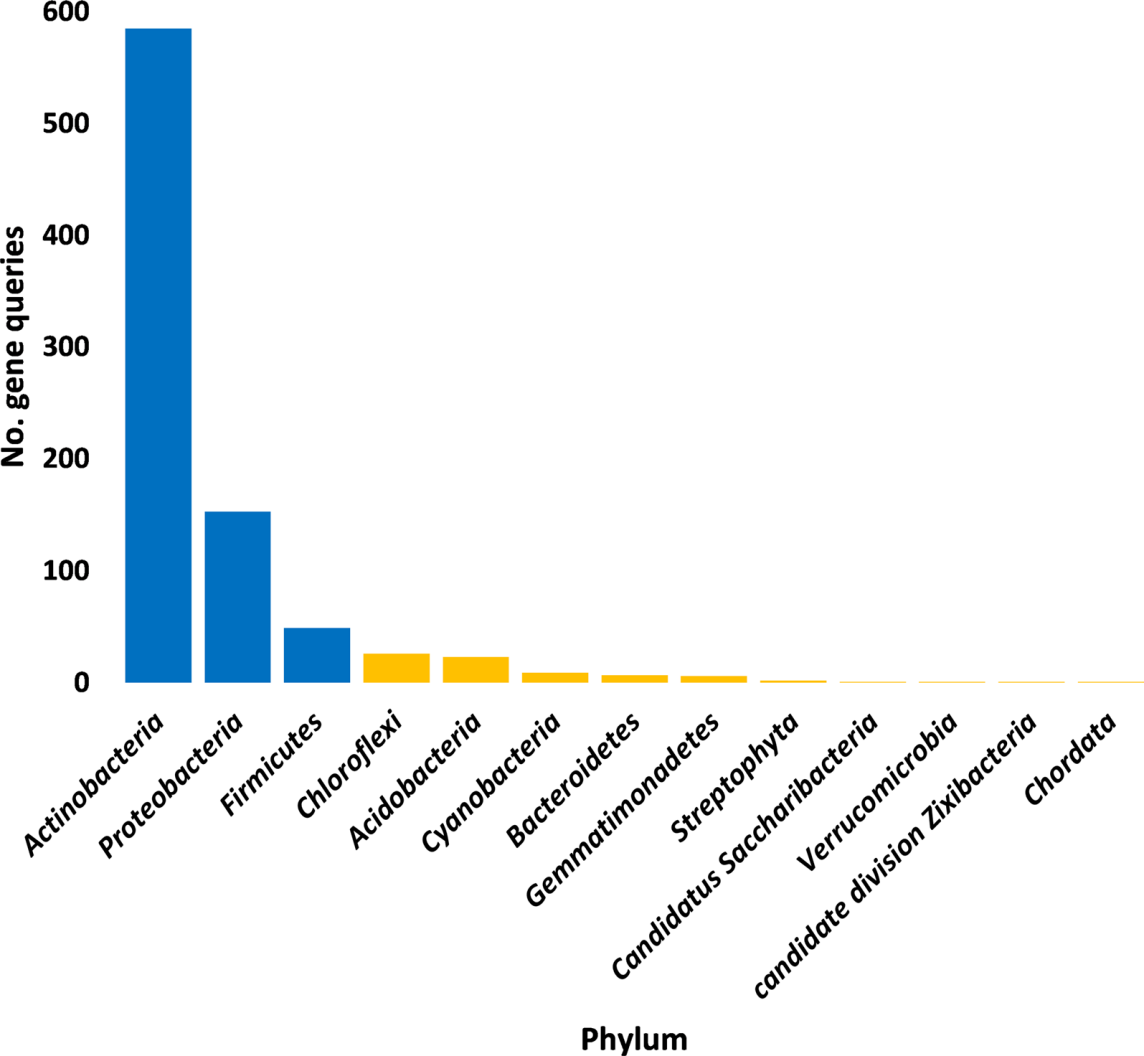
Query number of the most abundant (≥ 16) non-redundant antibiotic resistance genes (ARGs) of the antimicrobial resistance (AMR) families at the phylum level of soil microbiomes of *Abutilon fruticosum*. Orange columns refer to less abundant mechanisms that were not analyzed further. See Additional file 6: Table S6 for more details

**Fig. 3:**
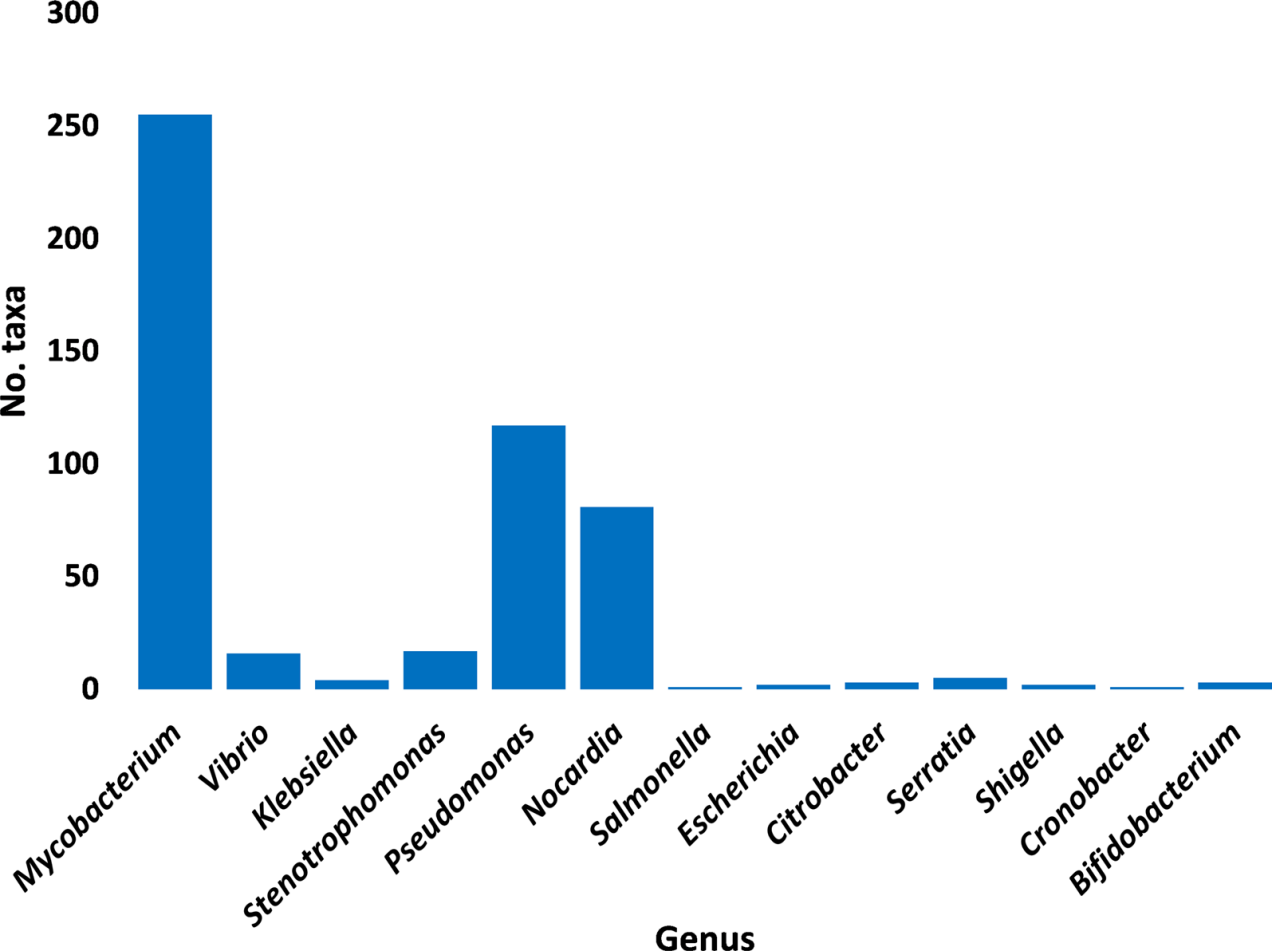
Number of taxa of bacterial genera in soil microbiomes of *Abutilon fruticosum* with the most abundant (≥ 16) non-redundant gene queries. Some taxa of these genera harbor species, whose ARGs are prevalent in human pathogens or bacterial colonizers (Additional file 4: Table S4). See Additional files 8, 9, 10, 11, 12, 13, 14, 15, 16, 17, 18, 19 and 20: Tables S8-20 for more information

**Fig. 4:**
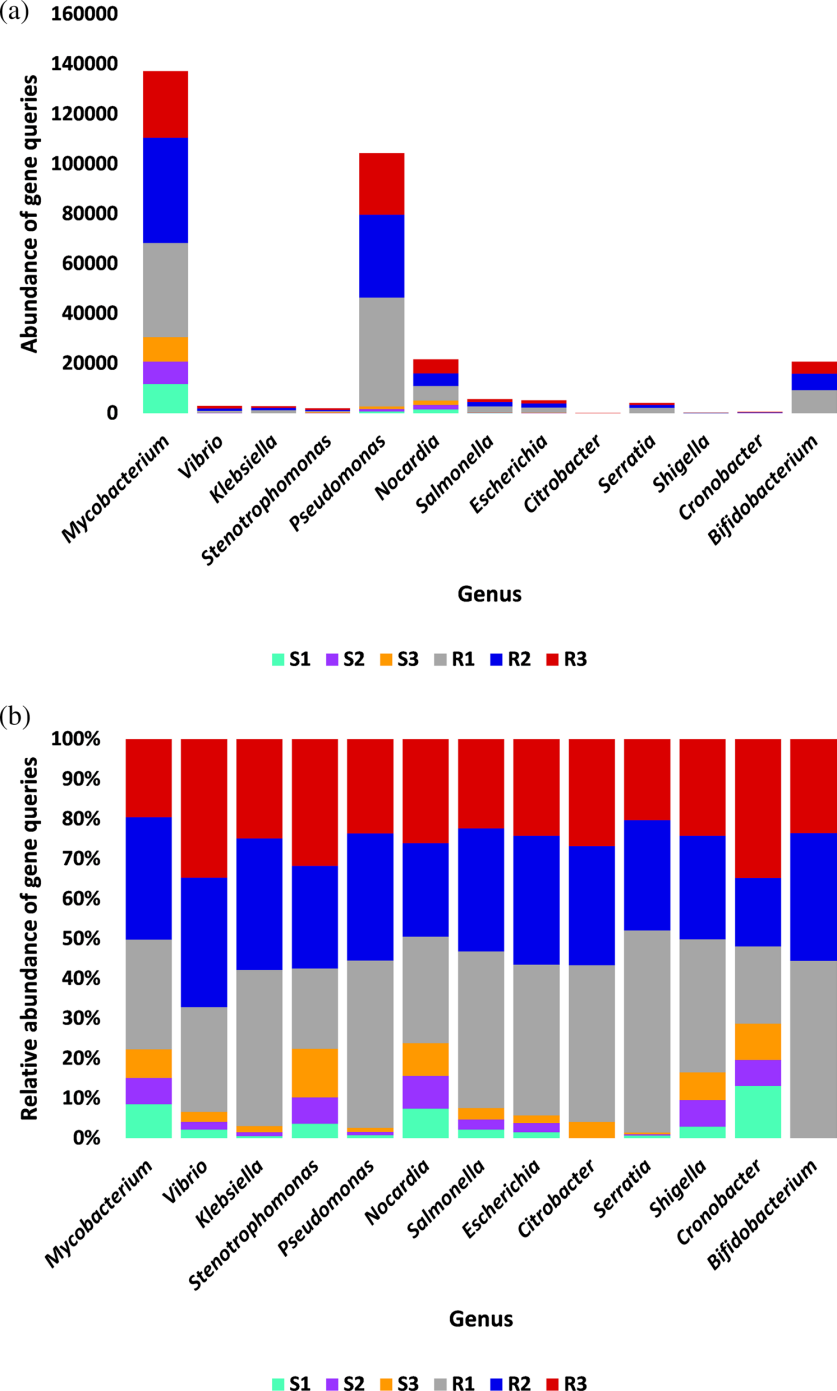
Abundance (**a**) and relative abundance (**b**) of the most abundant (≥ 16) non-redundant gene queries of bacterial genera in rhizosphere (R) and bulk (S) soil microbiomes of *Abutilon fruticosum*. These genera harboring species, whose ARGs are prevalent in human pathogens or bacterial colonizers, are shown in Additional file 4: Table S4. See Additional file 21: Table S21 for more information

A number of 16 antibiotic resistance genes (ARG) with > 15 non-redundant queries were detected (Additional file 3: Table S3). These ARGs were searched in CARD for prevalence in human pathogenic/colonizer bacteria (Additional file 4: Table S4) and those with no prevalence were not analyzed further. Based on this criterion, the number of ARGs for further analysis was narrowed to 10 (Fig. 1). The most abundant of which are *mtrA*, *soxR* and *vanRO* genes, while the least are *arr-1*, *ileS* and *iri* genes. List of ARGs in rhizospheric soil of *Abutilon fruticosum* that are prevalent in human bacterial pathogens/colonizers along with their phyla is shown in Additional file 5: Table S5. The selected microbes belong to the top two highly abundant phyla in soil rhizosphere of *Abutilon fruticosum* (Fig. 2 and Additional file 6: Table S6). These phyla are *Actinobaceria* and *Proteobacteria*. Genera of these phyla include *Mycobacterium*, *Nocardia* and *Bifidobacterium* of phylum *Actinobaceria*, and genera *Vibrio*, *Klebsiella*, *Stenotrophomonas*, *Pseudomonas*, *Salmonella*, *Citrobacter*, *Serratia*, *Shigella*, *Cronobacter* and *Escherichia* of phylum *Proteobacteria* (Additional file 5: Table S5). Bacterial genera/species that were not further examined because their ARGs are not common in human pathogens/bacterial colonisers include *Streptococcus lutetiensis* and *Staphylococcus haemolyticus* of the phylum *Firmicutes*. The results of abundance of non-redundant gene queries at bacterial genus/species level of rhizosphere (R) and bulk (S) soil microbiomes of *A. fruticosum* are shown in Additional file 7: Table S7, while abundance of individual genus/species with ARGs that are prevalent in human pathogens/bacterial colonizers and selected for further analysis are shown in Additional files 8, 9, 10, 11, 12, 13, 14, 15, 16, 17, 18, 19 and 20: Tables S8–S20. The number of taxa in these 13 selected genera is shown in Fig. 3. The highest number of taxa belongs to genera *Mycobacterium* (255), *Pseudomonas* (117) and *Nocardia* (81). The results for the 13 selected genera indicate higher abundance and relative abundance of ARG queries in rhizosphere soil of *A. fruticosum* compared with those of bulk soil (Fig. 4 and Additional file 21: Table S21). The highest abundance average across soil type was detected for genera *Mycobacterium* (22873), *Pseudomonas* (17378), *Nocardia* (3607) and *Bifidobacterium* (3452) (Fig. 4a).

**Fig. 5:**
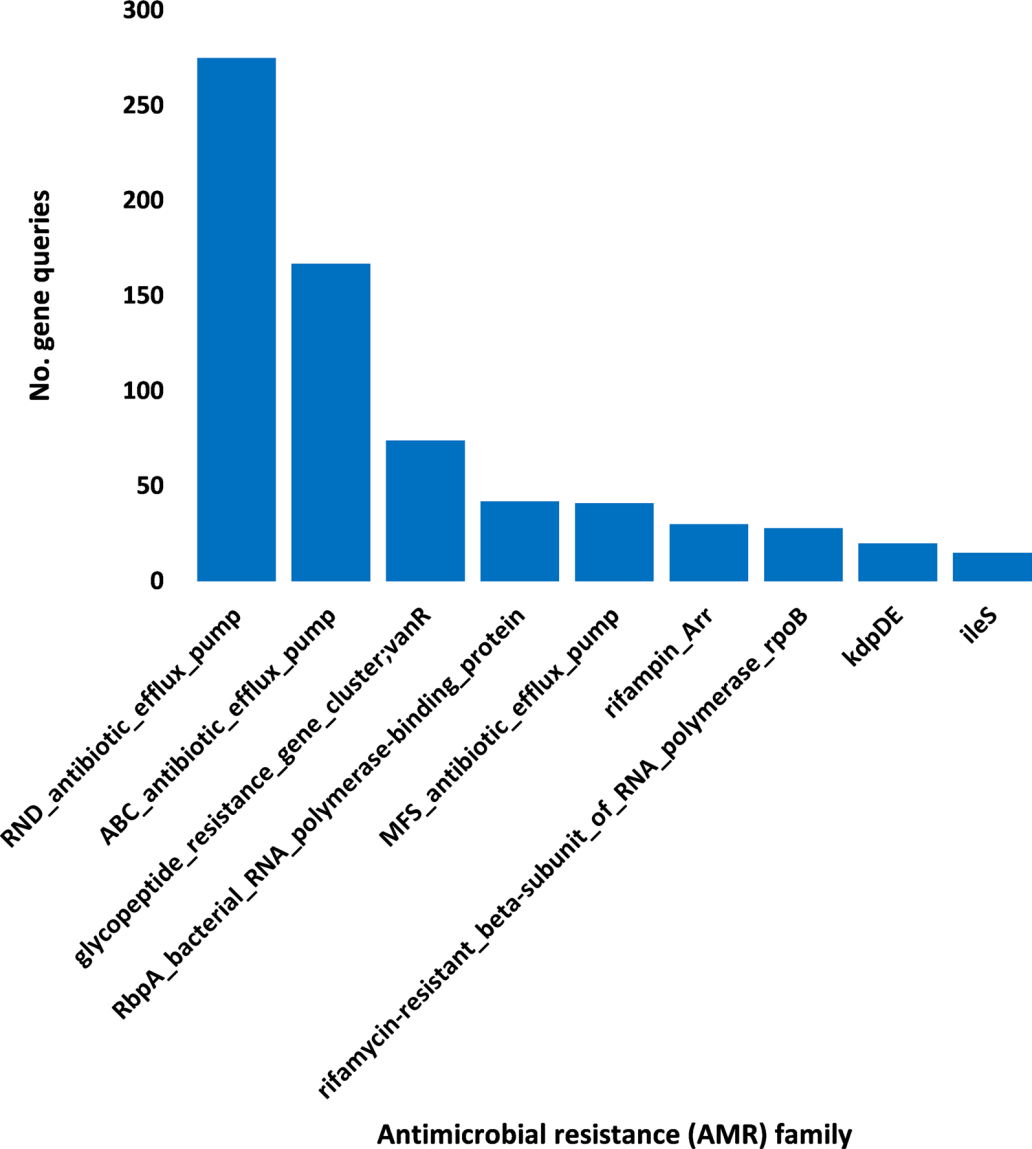
Query number of the most abundant non-redundant antibiotic resistance genes (ARGs) of the antimicrobial resistance (AMR) families at the phylum level of soil microbiomes of *Abutilon fruticosum*. See Additional file 6: Table S6 for more information

Description of antimicrobial resistance (AMR) families along with resistance mechanisms of the 10 selected ARGs are shown in Additional file 22: Table S22. The most highly abundant resistance families (≥ 15) are shown in Table S6 and described in Fig. 5. Resistance mechanisms of these families include antibiotic efflux pump (four families), antibiotic target alteration (three families), antibiotic target protection (one family) and antibiotic inactivation (one family) (Additional file 22: Table S22). AMR families of the resistance mechanism of antibiotic efflux pump include resistance-nodulation-cell division (RND) antibiotic efflux pump (for *mtrA*, *soxR* and *golS* genes), major facilitator superfamily (MFS) antibiotic efflux pump (for *soxR* gene), the two-component regulatory kdpDE system (for *kdpE* gene) and ATP-binding cassette (ABC) antibiotic efflux pump (for *efrA* gene). AMR families of the resistance mechanism of antibiotic target alteration include glycopeptide resistance gene cluster (for *vanRO* gene), rifamycin-resistant beta-subunit of RNA polymerase (for *rpoB2* gene) and antibiotic-resistant isoleucyl-tRNA synthetase (for *ileS* gene). AMR families of the resistance mechanism of antibiotic target protection include bacterial RNA polymerase-binding protein (for *RbpA* gene), while those of the resistance mechanism of antibiotic inactivation include rifampin ADP-ribosyltransferase (for *arr-1* gene) (Additional file 22: Table S22).

**Fig. 6:**
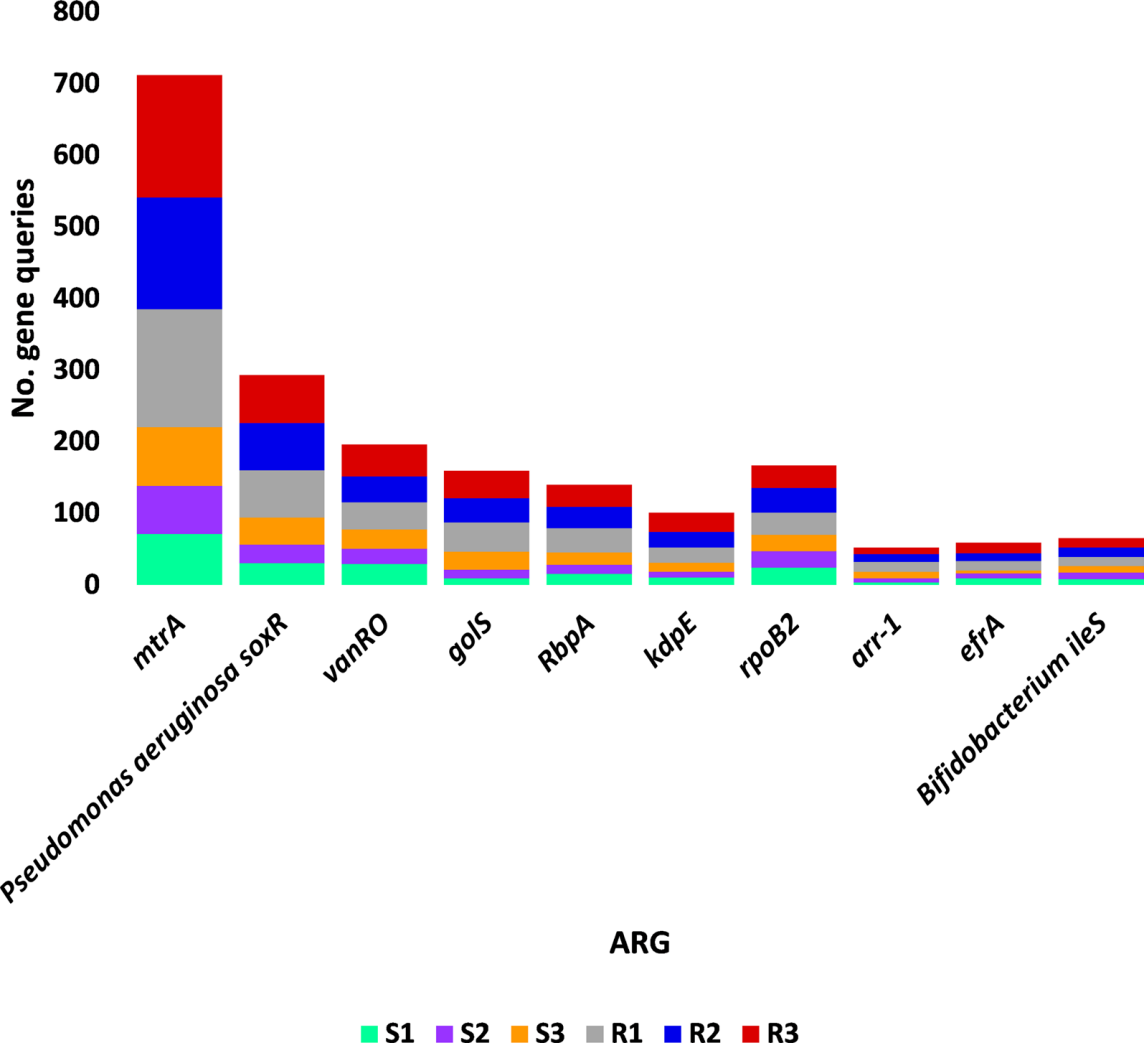
Query number of the most abundant (≥ 16) non-redundant antibiotic resistance genes (ARGs) of rhizosphere (R) and bulk (S) soil microbiomes of *Abutilon fruticosum*. See Table Additional file 23: S23 for more details

**Fig. 7:**
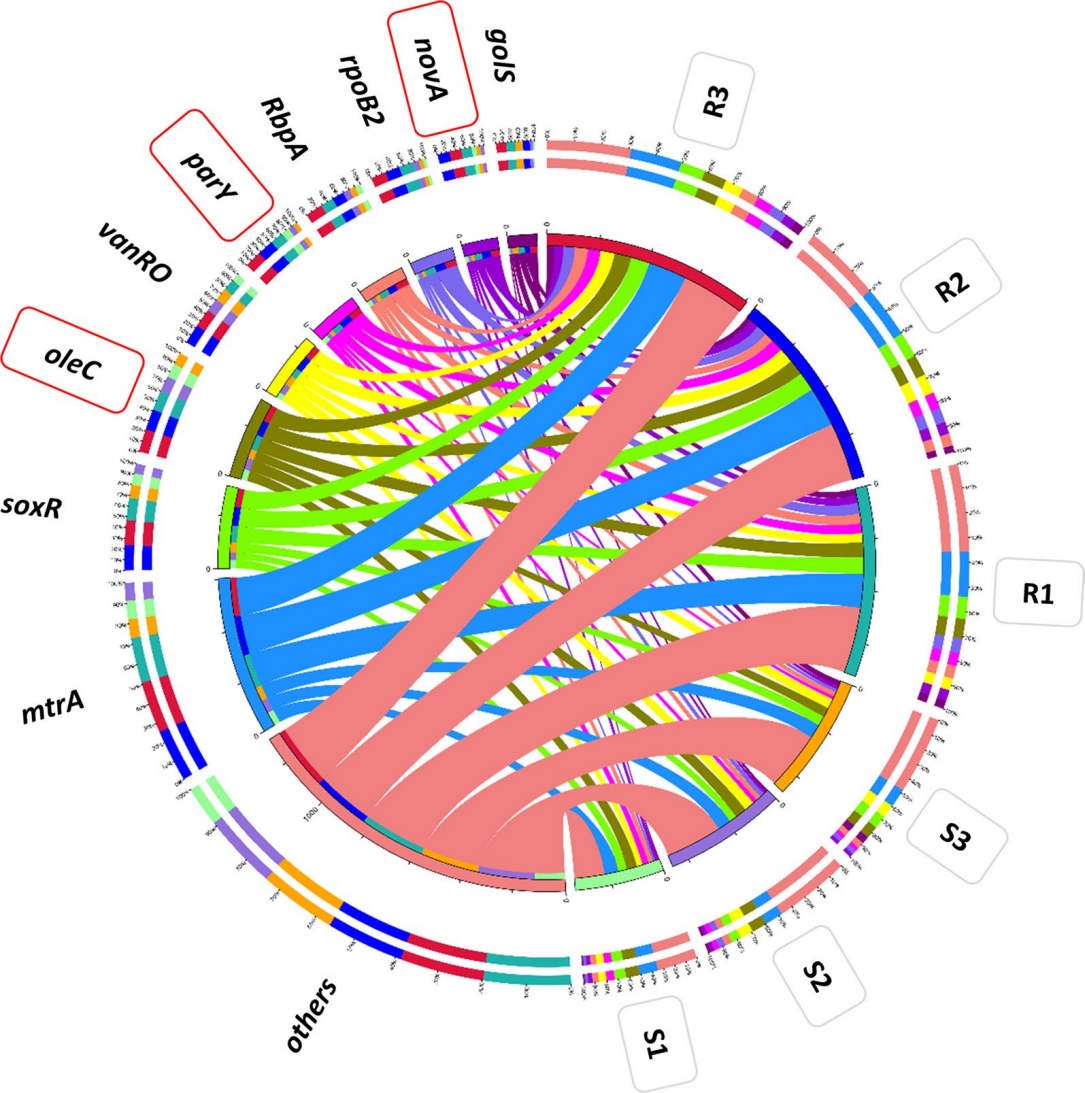
Circle chart of the top highly abundant antibiotic resistance genes (ARGs) in rhizosphere (R) and bulk (S) soil microbiomes of *Abutilon fruticosum*. The chart is divided into two sides, where the right one refers to samples (S & R) information and the left refers to antibiotic resistance genes (ARGs) information. The wideness of different scales of inner and outer circles refers to abundances of ARGs in different soil samples. Colors in inner circle refer to sum of abundance of all ARGs per sample (right side) and sum of abundance of all samples per ARG (left side). Colors in outer circle refer to detailed abundance of different ARGs per sample (right side) and detailed abundance of different samples per ARG (left side). Red boxes refer to ARGs that are not prevalent in human pathogens or bacterial colonizers, thus, not analyzed further. See Additional file 24: Table S24 for more information

**Fig. 8:**
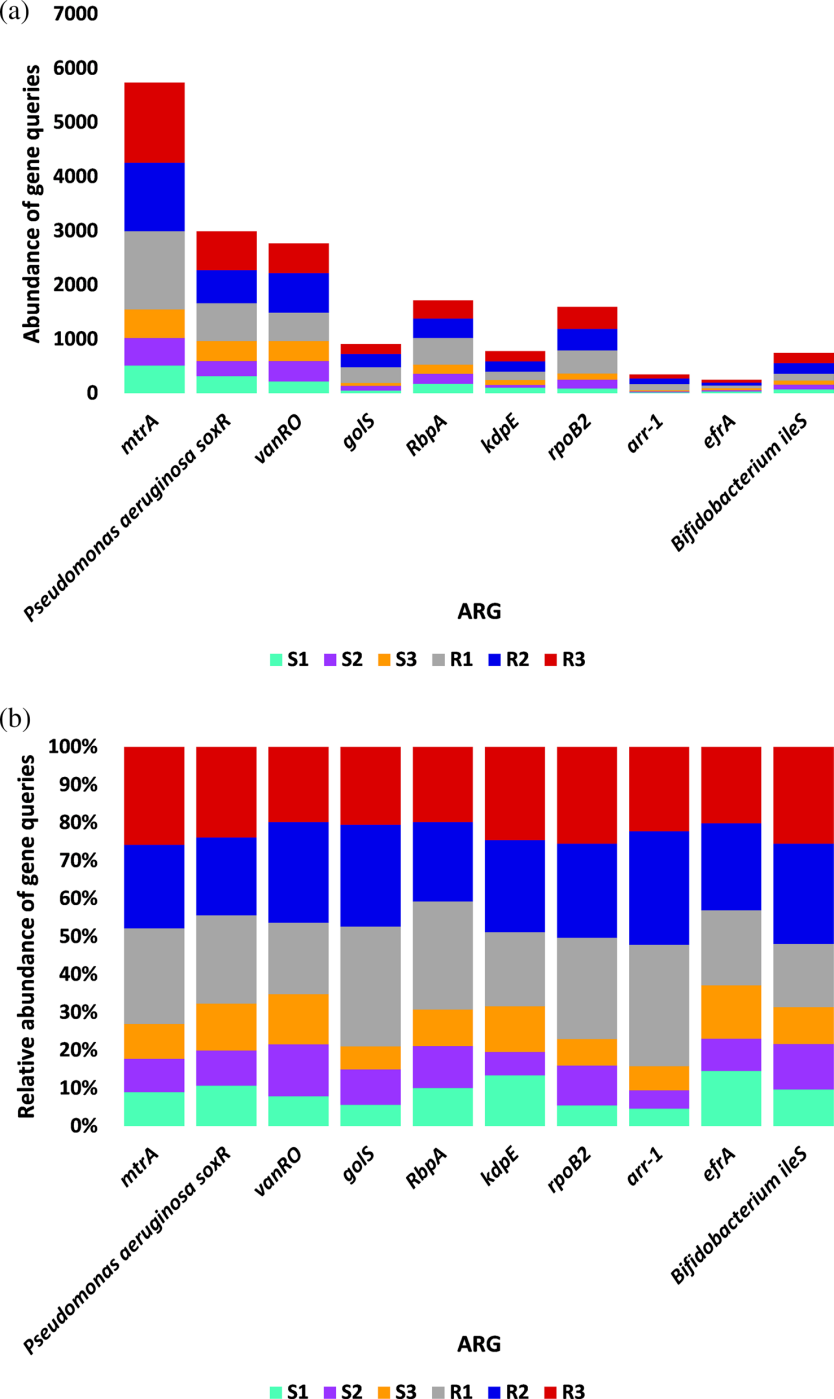
Abundance (**a**) and relative abundance (**b**) of the most abundant (≥ 16) non-redundant antibiotic resistance gene queries (ARGs) of rhizosphere (R) and bulk (S) soil microbiomes of *Abutilon fruticosum*. See Additional file 24: Table S24 for more information

The results for the number of non-redundant queries of ARGs prevalent in human pathogens/bacterial colonizers at genus/species level in rhizosphere (R) and bulk (S) soil microbiomes of *Abutilon fruticosum* are shown in Additional file 23: Table S23 and described in Fig. 6, while the results of ARG abundance are shown in Additional file 24: Table S24 and described in Figs. 7 and 8. The results in Fig. 7 refer on the six most abundant ARGs, while those of Fig. 8 refer to the 10 most abundant ARGs. The highest abundance average across soil type was detected for *mtrA* (3452), *soxR* (498), *vanRO* (461), *RbpA* (286) and *rpoB2* (266) (Fig. 8a). Overall, the number, abundance and relative abundance of ARG in the rhizosphere soil of *A. fruticosum* were higher than those in the bulk soil (Figs. 6, 7 and 8).

## Discussion

The extensive use of antibiotics across a number of decades has led to the selection of resistant bacterial strains to almost all antibiotics (Peterson and Kaur 2018). Although metagenomic resistance determinants seem to pose no threat to human health, they can be mobilized to indigenous plasmids and/or integrons and be horizontally transferred to bacterial clinical isolates, then, an outbreak can occur. Such speculation can be promoted by the selective pressure initiated by human activities that can result in the enrichment of these determinants and self-resistance in members of the interacting bacterial community due to high plasticity of bacterial genomes and high potential for adaptability (Verma et al. 2019). Interestingly, selective pressure can further mediate the induction of bacterial cell competence and membrane permeability (Prudhomme et al. 2006). Another hypothesis was set to describe the approach of mediating emergence of antibiotic-resistance microbes (Surette and Wright 2017). It suggests that antibiotics provide a strong selection to capture antibiotic resistance genes by mobile genetic elements (MGEs) that eventually can, as well, promote bacterial cell competence. Examples of these bacterial MGEs include those of *Streptococcus pneumonia* (Prudhomme et al. 2006) and *Staphylococcus aureus* (Goerke et al. 2006). Furthermore, antibiotics were recently reported to affect accidental packaging of ARGs into phage particles (Stanczak-Mrozek et al. 2017) and extensive exposure to antibiotics likely results in increased rates of genetic mutation and recombination (Blázquez et al. 2012).

Acquisition of antibiotic resistance by pathogenic bacteria is envisioned to occur sequentially starting from emergence of the ARG followed by mobilization, transfer to pathogens/bacterial colonizers, dissemination, and possibly transfer of resistance from pathogens/bacterial colonizers to environmental bacteria or opportunistic pathogens (Bengtsson-Palme 2018; Peterson and Kaur 2018). Such events of genetic exchange are likely to be promoted under certain settings (or hot-spots) such as sewage, hospital effluents, aquaculture, colonized human or animal host, gut microbiome, and biofilms, where density of bacteria and mobile elements in these settings is high (Modi et al. 2013; Resch et al. 2005; Stanczak-Mrozek et al. 2015; Von Wintersdorff et al. 2016). Mobile genetic elements (MGEs), e.g., viromes or plasmidomes, are main reasons for rapid dissemination of antibiotic resistance genes within closely-related or even genetically-distant microbes in the environment via horizontal gene transfer (HGT) (WHO 2012). During the food chain, it is unlikely to completely avoid dissemination of contaminated food with microbes harboring antibiotic resistance genes (ARGs) in different packages, e.g., mobile genetic elements (MGEs). Thus, ARGs can horizontally pass into human gut microbiome (Chen et al. 2017; 2019). However, limitations to the occurrence of genetic exchange include the narrow host range of MGEs, existence of the restriction/modification system in bacteria, the high fitness cost of integrating a new gene, and the inability of MGEs to persist in the new environment (Domingues et al. 2012).

### Resistance mechanisms in rhizospheric soil resistome of Abutilon fruticosum

Among known resistance mechanisms in bacteria (Munita and Arias 2016; Peterson and Kaur 2018; Wilson et al. 2020), those that are highly abundant in rhizospheric soil microbiome of *Abutilon fruticosum* (Additional file 24: Table S24) mainly include antibiotic efflux pump (four antimicrobial families), antibiotic target alteration (three families), antibiotic target protection (one family) and antibiotic inactivation (one family) (Additional file 22: Table S22). Mode of action of ARGs of the different mechanisms and antimicrobial resistance families and expected consequences are discussed below. These ARGs exist in bacterial genera with species harboring ARGs that are prevalent in human pathogens/bacterial colonizers (Additional file 4: Table S4 and Additional file 9, 10, 11, 12, 13, 14, 15, 16, 17, 18, 19 and 20: TablesS9–S20).

1. Antibiotic efflux pump mechanism of resistance

As soon as bacterial cells are exposed to specific toxic substances or antibiotics, they immediately start expulsing them by an active transport system, which eventually help bacteria acquire multidrug resistance (Fernández and Hancock 2012). For human pathogens, antibiotic efflux mechanism should be stronger and faster than the host defense mechanism or antibiotic therapy (Koprivnjak and Peschel 2011) or otherwise, bacteria will fail to continue growth. Known bacterial efflux pump families include resistance-nodulation-cell division (RND), ATP-binding cassette (ABC) family, major facilitator superfamily (MFS), two-component regulatory system (TCS) of kdpDE, small multidrug resistance (SMR) family and multidrug and toxic compound extrusion (MATE) family (Cardona et al. 2018; Piddock 2006; Poole 2007; Sun et al. 2014; Tierney and Rather 2019). The different efflux pumps are energy-dependent. Of which, ABC uses ATP for activation, while other pumps use proton motive forces (Chitsaz and Brown 2017).

In the rhizospheric soil of *Abutilon fruticosum*, four AMR families of antibiotic efflux pump were detected*.* They are RND that includes *mtrA*, *soxR* and *golS* genes, MFS that includes *soxR* gene, two-component regulatory system of kdpDE that includes *kdpE* gene and ABC that includes *efrA* gene. These four efflux processes of RND, ABC, MFS and TCS-KdpDE are the most highly abundant in rhizospheric soil microbiome of *A. fruticosum* (Fig. 5). These mechanisms are activated due to the high abundance of ARGs *mtrA*, *soxR*, *golS*, *kdpE* and *efrA* (Figs. 7 and 8). The *mtrA* gene belongs to members of phyla *Actinobacteria* (genus *Mycobacterium*) and *Proteobacteria* (genus *Vibrio*), while *soxR* gene belongs to members of phylum *Proteobacteria* (genera *Klebsiella*, *Stenotrophomonas* and *Pseudomonas*), *golS* gene belongs of members of phylum *Proteobacteria* (genus *Salmonella*), *kdpE* gene belongs to members of phyla *Actinobacteria* (genus *Mycobacterium*) and *Proteobacteria* (genera *Citrobacter*, *Escherichia*, *Klebsiella*, *Pseudomonas*, *Salmonella*, *Serratia*, *Shigella* and *Stenotrophomonas*), and *efrA* gene belongs to members of phylum *Proteobacteria* (genera *Cronobacter* and *Klebsiella*) (Additional file 5: Table S5).

The RND superfamily transporters refer to large polypeptide chains that are involved in maintaining cell homeostasis and in expelling a large number of toxic compounds and virulence determinants (Coyne et al. 2010). The *mtrA* (multiple transferable resistance A) gene that is highly abundant in rhizospheric microbiome of *A. fruticosum* (Fig. 8) belongs to the two-component mtrAB system of RND family (Begmatov et al. 2022; Qin et al. 2022). In general, the two-component system (TCS) is considered as the main sensor/responder for environmental adaptation (Qin et al. 2022) as it responds to the presence of antibiotics, and participates in the realm of infectious diseases caused by pathogenic bacteria (Cardona et al. 2018; Tierney and Rather 2019). The mtrAB system is highly conserved among members of phylum *Actinobacteria*, like those of genus *Mycobacterium*, and acts, not only in bacterial cell wall homeostasis, but also in cell shape and proliferation, in osmoprotection, and in antibiotic resistance (Hoskisson and Hutchings 2006; Qin et al. 2022). The *mtrA* regulon was recently proven to participate in cell division, and in cell wall metabolism in members of genus *Mycobacterium* (Gorla et al. 2018). This response regulator also confers resistance against rifampicin and vancomycin and tolerance against different types of cell envelope stresses (Gorla et al. 2018; Qin et al. 2022). MtrA also participates in the construction of the bacterial multiple transferable resistance (MTR) complex that accelerates the energy-dependent MtrCDE efflux pump (Nikaido 1996; Rouquette et al. 1999). This pump also serves in expelling several other antibiotics, like penicillin, erythromycin, cephalosporin and rifampin (Handing et al. 2018; Olesky et al. 2006). The rhizospheric soil microbiome of *A. fruticosum* seems to lack the *mtrR* gene (Additional file 3: Table S3), which promotes sensitivity against hydrophobic agents (HAs) or antibacterial peptides (Handing et al. 2018) by encoding a transcriptional repressor of MtrCDE multidrug efflux pump system (Lucas et al. 1997). As members of the MtrCDE efflux pump system exist in the rhizospheric resistome of *A. fruticosum* (Additional file 3: Table S3), while *mtrR* gene is absent, then, it is likely that this resistance system can act effectively in members of genus *Mycobacterium*. In terms of *Vibrio cholerae* of phylum *Proteobacteria*, little is known about its utilization of the mtrAB system against antibiotics. Rather, it utilizes the WigK/R TCS, a histidine kinase/response regulator, which confers cell envelope homeostasis as a response to cell wall stress induced by different antibiotics, e.g., β-lactams (Dörr et al. 2016).

The *soxR* gene is a transcription factor that acts as a global response modulator of bacterial multidrug resistance and fitness (Li et al. 2017; Palma et al. 2005). SoxR was proven to regulate several RND efflux pump genes including SoxR/SoxS and adeB/J/G systems (Gu and Imlay 2011; Marchand et al. 2004). The *soxR* gene contributes to antibiotic resistance in members of genus *Pseudomonas* of phylum *Proteobacteria* (Palma et al. 2005; Sakhtah et al. 2016). Due to the gene’s participation in the SoxR/SoxS paradigm, oxidized SoxR protein further oxidizes its other component namely SoxS (Li et al. 2017; Pomposiello and Demple 2001). SoxS, in turn, promotes expression of the downstream multidrug efflux tripartite transporter system namely AcrAB-TolC (Ruiz and Levy 2014; White et al. 1997). Moreover, SoxR, alone, can directly regulate an efflux pump of 6-gene regulon in *Pseudomonas aeruginosa* and in the human pathogen *Actinetobacter baumannii* (Bialek-Davenet et al. 2011; Li et al. 2017; Peleg et al. 2008). The two genes of the SoxR/SoxS system exist in the rhizospheric soil microbiome of *A. fruticosum* (Additional file 3: Table S3) of which *soxR* is highly abundant (Fig. 8 & Additional file 24: Table S24). Moreover, SoxR also acts as a mediator of MFS efflux pump (Dulyayangkul et al. 2016; Saidijam et al. 2006). This system refers to several membrane transport proteins that facilitate re-movement of uptaken drugs across membranes, while block bidirectional passage across the membrane as a consequence of the chemiosmotic gradient (Abramson et al. 2003; Marger and Saier 1993).

Other members of the RND efflux system include *golS* gene that encodes a mercury resistance (MerR)-like sensor (Brown et al. 2003; Pérez Audero et al. 2010). MerR was proven to regulate two proteins namely GolT (a P-type ATPase) and GolB (a small cytoplasmic metal-binding RND protein of the inner membrane) (Pontel et al. 2007). These two proteins participate in RND-dependent efflux system to promote resistance against gold salts (Au) inside the poisonous bacteria *Salmonella*. This bacteria dwells contaminated water or food to infect the human intestinal tract. The higher the GolS concentration, the higher the sequestration rate of intracellular free Au (Brown et al. 2003). With high Au levels, an efflux system namely CBA is induced. This efflux system is a complex of three subunits C, B and A that promote efflux of drugs from the cytoplasm or from the periplasm (Brown et al. 2003). The *golS* gene is highly abundant in the rhizospheric soil microbiome of *A. fruticosum* (Fig. 8 and Additional file 24: Table S24).

The two-component system of KdpDE also exists in *Salmonella* dwelling in rhizospheric soil of *A. fruticosum* (Additional file 3: Table S3). It is made of a histidine kinase (HK) protein that senses environmental signals, and a response regulator (RR) that mediates cellular response, thus, alter expression of downstream target genes (Freeman et al. 2013). These two proteins form the two-component regulatory complex system of KdpD (HK)/KdpE (RR) that regulates the potassium dependent- (Kdp-) ATPase pump of the bacterial operon KdpFABC (Wang et al. 2019). The KdpD/KdpE system was reported to increase the ability of bacteria to survive in the human host cells and cause the disease (Alegado et al. 2011). KdpD, in particular, was proven to maintain bacterial growth in macrophage of nematode cell lines, while KdpE acts as a response regulator in conferring resistance to the antibiotic kanamycin in *E. coli* (Hirakawa et al. 2003; Lv et al. 2021). This protein also mediates expression of virulence gene in a specific member of the genus *Escherichia* namely *Enterohaemorrhagic E. coli* (EHEC) (Alegado et al. 2011; Hughes et al. 2009; Xue et al. 2011). This expression results in the increased survival rate of bacteria in human macrophages. The genes encoding KdpD and KdpE exist in the rhizospheric soil of *A. fruticosum* (Additional file 3: Table S3) of which the latter is highly abundant (Additional file 24: Table S24).

The ATP-binding cassette (ABC) family is made of membrane and membrane-associated (e.g., AAA ATPases) proteins that transports drugs extracellularly. In the ABC system, ATPases hydrolyze ATP to release energy required for the translocation (efflux) of substrates or drugs across membranes (Sauna et al. 2009). Of which, the TCS of efrAB participates as a heterodimeric ABC multidrug efflux pump in *Enterococcus faecalis* (García-Solache and Rice 2019; Shiadeh et al. 2019) that can cause drug resistance when *efrA* and *efrB* genes are concurrently expressed (Lubelski et al. 2007). The erfAB system is enriched by the presence of several antibiotics including gentamicin, streptomycin and chloramphenicol (Lerma et al. 2014). These two genes exist in rhizospheric soil of *A. fruticosum* (Additional file 3: Table S3), of which, *erfA* gene is highly abundant (Additional file 24: Table S24).

2. Antibiotic target alteration

AMR families of the resistance mechanism of antibiotic target alteration include glycopeptide resistance gene cluster (for *vanRO* gene), rifamycin-resistant beta-subunit of RNA polymerase (for *rpoB2* gene) and antibiotic-resistant isoleucyl-tRNA synthetase (for *ileS* gene) (Additional file 22: Table S22). These three genes are highly abundant in rhizospheric soil microbiome of *A. fruticosum* (Additional file 24: Table S24). This resistance mechanism refers to the ability of the bacteria to modify the 3D structure of its membrane receptors to reduce their affinity for antibiotics. In terms of *vanRO* gene, the most common example is the glycopeptides (ex., vancomycin) that kill bacteria by inhibiting synthesis of cell wall (Munita and Arias 2016). Vancomycin resistance is common in *Enterococcus faecium* of phylum *Firmicutes*, a pathogenic microbe that causes human diseases, like neonatal meningitis or endocarditis (Arias and Murray 2012). This microbe does not exist in rhizospheric soil resistome of *A. fruticosum*, but the encoding gene, e.g., *vanRO*, was more recently confirmed to exist in genera of phylum *Actinobacteria*, e.g., *Mycobacterium* and *Nocardia* (Additional file 5: Table S5). Resistance against vancomycin is conferred by one or more of the 11 *van* operons that were proven previously to be horizontally transferred via MGEs, e.g., conjugative or non-conjugative plasmids carrying Tn*3* transposons, to clinical isolates of *E. faecium* (Arias and Murray 2012; Munita and Arias 2016). The *vanO* operon includes two gene clusters namely *vanSO*/*vanRO* and *vanHOX* in addition to five ORFs, e.g., ORFs1-3 and ORFsA-B, with unknown function (Gudeta et al. 2014). The cluster *vanSO*/*vanRO* acts as a two-component system, where VanSO is the histidine kinase (HK) and VanRO is the response regulator (RR). The *vanHOX* gene cluster exists head-to-head to the *vanSO*/*vanRO* gene cluster. The VanSO/VanRO cluster promotes biosynthesis of the VanHOX cluster in two steps, where VanS senses the accumulation of substrates in order to inhibit glycosyltransferase activity, then VanR is activated by ATP-dependent phosphorylation. VanR, then, initiates expression of the *vanHOX* gene cluster, where *vanH* gene encodes a dehydrogenase enzyme that participates in the biosynthesis of new peptidoglycan precursors, while *vanX* gene encodes an enzyme that removes the normal D-Ala-D-Ala-ending precursors, and *vanO* gene encodes a ligase to synthesize a new altered D-Ala-D-Lac substrate of penicillin binding protein (PBP) with low binding affinity of vancomycin (Gudeta et al. 2014; Munita and Arias 2016; Reynolds 1989). *vanRO* and *vanSO* genes are highly abundant in rhizosphere soil of *A. fruticosum* (Additional file 24: Table S24)*.*

The antibiotic resistance gene *rpoB2* was previously proven to exist in members of the environmental saprophytes of genus *Nocardia*, e.g., pathogens that cause the infectious disease nocardiosis (Ishikawa et al. 2006). Genome of this genus contained two genes, namely *rpoB* and *rpoB2*, encoding RNA polymerase (RNAP) β subunit and share 88.8% identity*.* RpoB-encoded protein is rifampin sensitive, whereas RpoB2-encoded protein contains amino acid substitutions at the rifampin-binding site that enable bacteria to resist this antibiotic (Severinov et al. 1993). Rifampin is considered as a front-line drug for the treatment of several other infectious diseases including tuberculosis and resistance to rifampin in clinical isolates is likely to be due to mutations resulting in ≥ 8 amino acid substitutions in the *rpoB* gene to generate the *rpoB2* gene. Moreover, members of genus *Nocardia* possess a number of rifampin-inactivating enzymes as an extra type of resistance in this bacterium. The *rpoB2* gene is highly abundant in rhizosphere soil microbiome of *A. fruticosum* (Additional file 3: Table S3)*.*

In terms of the antibiotic resistance gene *ileS* (encoding isoleucyl-tRNA synthetase or IleS) of *Bifidobacteria*, it confers resistance against the narrow-spectrum antibiotic mupirocin that is firstly discovered in *Pseudomonas fluorescens* (Serafini et al. 2011; Sutherland et al. 1985). *Bifidobacteria* are natural inhabitants of the mammalian gastrointestinal tract that act in maintaining gastrointestinal health. The aminoacyl-tRNA synthetase enzymes originally act in catalyzing the aminoacylation of tRNA by their cognate amino acids. Mode of action of the antibiotic mupirocin is competing with isoleucine as a substrate for isoleucyl-tRNA synthetase (Hughes and Mellows 1980), thus, all polypeptide chains will stop elongation at the codon of isoleucine at the presence of the antibiotic. Resistance to mupirocin is based on sequence variability of the *ileS* gene and its encoded enzyme. Originally, the IleS enzyme contains a reactive site, e.g., the binding site of Ile-AMP, during the aminoacylation process, while the hydrophobic valine residue serves for the stability of the ligand during aminoacylation, thus the possibility to bind the antibiotic. In *Bifidobacteria*, this amino acid residue is replaced for a tyrosine’s hydrocarbon benzene ring, which results in the reduced stability of the ligand in the active site, thus, release of the antibiotic. The *ileS* gene is highly abundant in rhizosphere soil of *A. fruticosum* (Additional file 24: Table S24)*.*

- 3.Antibiotic target protection

The *RbpA* gene encodes a multi-functional RNAP-binding protein that provide resistance mechanisms of target protection and target alteration in members of the genus *Mycobacterium* (Additional file 5: Table S5). Among these functions, RbpA acts as a stimulator of the core RNA-polymerase (RNAP), e.g., σ^A^ activity, or as a transcription activator of the enzyme (Hu et al. 2012). RbpA also assists in the expression of virulence genes and in proliferation of human pathogens (Hu et al. 2012). When the antibiotic rifampicin binds its target site in RNAP, it prevents association of σ^A^ to RNAP, thus, inhibits transcription of bacterial genes. The *rbpA* gene was reported to be highly upregulated during rifampicin treatment (Maeda et al. 2000; Paget et al. 2001). The binding site of RbpA in RNAP can be the exact target of the antibiotic, thus, when the RbpA protein exists in excessive amount, it can occupy the antibiotic’s target site and make the site inaccessible to the antibiotic (Newell et al. 2006; Wilson et al. 2020). Furthermore, RbpA can bind to cluster I of the β subunit of RNAP, which results in the alteration of the enzyme’s 3D structure and its antibiotic target site. This conformational change can prevent association of the antibiotic to its target site of the enzyme (Hu et al. 2012). The *rbpA* gene is highly abundant in rhizosphere soil of *A. fruticosum* (Additional file 24: Table S24)*.*

- 4. Antibiotic inactivation

The *arr-1* gene encodes rifampin ADP-ribosyltransferase that confers the resistance mechanism of antibiotic inactivation against rifampin in members of genus *Mycobacterium* (Additional file 5: Table S5). The enzyme acts in catalyzing ADP-ribosylation of the antibiotic in order to inactivate it (Morgado et al. 2021). As indicated earlier, mode of action of this antibiotic is binding to the B subunit of the RNA polymerase in order to inhibit transcription of bacterial genes. The antibiotic inactivation resistance mechanism by ADP-ribosylation governed by *arr-1* gene refers to the generation of a modified, inactive structure of the antibiotic, while mechanism of antibiotic target protection for the *rbpA* gene refers to the generation of a modified version of the antibiotic’s target site in the enzyme. The *arr-1* gene exists in the bacterial chromosome, hence, cannot be incorporated in bacteriophages except by accidental packaging (Wang et al. 2018). This gene is highly abundant in rhizosphere soil of *A. fruticosum* (Additional file 24: Table S24)*.*

Based on the results of the present study, there are two types of bacteria that exist in the rhizospheric soil of *A. fruticosum* and can be candidates for posing threats to human health. The first type refers to the human commensal colonizer bacteria of the different genera of phyla *Actinobacteria* and *Proteobacteria* that harbor abundant ARGs in rhizospheric soil microbiome of *A. fruticosum*. The second type refers to human pathogens of the same two phyla existing in the rhizospheric soil microbiome of *A. fruticosum*, whose abundant ARGs are prevalent. The commensal colonizer microbe type can be dangerous if their ARGs are transferred via one or more of the mechanisms of horizontal gene transfer (HGT) via food chain to pathogens or human clinical isolated in gut. These commensal bacteria can also be converted to opportunistic pathogens when they incorporate abundant ARGs in their cells. Risk to human health can be larger if these ARGs are prevalent in human pathogens (or opportunistic pathogens) that already exist in rhizosphere soil microbiome of *A. fruticosum.* These types of bacteria can be a direct threat to human health without the need to be horizontally transferred to genetically-related or -unrelated bacteria.

In conclusion, the present study provides insights into the signature and possible horizontal transfer and/or dissemination of rhizospheric resistome determinants of the wild plant *A. fruticosum* to the environment and possible threat to human health. Attention should also be given to rhizobiome signature of different wild plant species that can help isolate new antibiotics with feasible therapeutic interventions.

### Supplementary Information

**Additional file 1.** **Table S1**. Alignment results of non-redundant gene queries generated from rhizosphere (R) and bulk (S) soil microbiomes of *Abutilon fruticosum*. NOVO_MIX refers to reassembled low-copy ORF that is usually found in ≥ 2 of the six microbiome samples, while that preceded by S or R refers to a unique ORF.

**Additional file 2.** **Table S2**. Annotation results of non-redundant queries of antibiotic resistance genes (ARG) generated from rhizosphere (R) and bulk (S) soil microbiomes of *Abutilon fruticosum*. NOVO_MIX refers to reassembled low-copy ORF that is usually found in ≥ 2 of the six samples, while that preceded by S or R refers to a unique ORF.

**Additional file 3.** **Table S3**. Number and description of non-redundant queries of antibiotic resistance genes (ARG) generated from rhizosphere (R) and bulk (S) soil microbiomes of *Abutilon fruticosum*. NOVO_MIX refers to reassembled low-copy ORF that is usually found in ≥ 2 of the six samples, while that preceded by S or R refers to a unique ORF. ARGs with query no. of ≤ 15 (in red) were not analyzed further.

**Additional file 4.** **Table S4**. Pathogens or bacterial colonizers harboring antibiotic resistance genes (ARGs) that are prevalent in human. The data were generated using the resistance gene identifier (RGI) that is a tool for putative AMR gene detection from submitted sequence annotation data in the comprehensive antibiotic resistance database (CARD). Species in red refers to a microbe acting as a biocontrol agent and later was placed into the low risk group 1 (classification according to Technical Rules for Biological Agents (TRBA) from the German Federal Institute for Occupational Safety and Health, TRBA 466 “Classification of prokaryotes (bacteria and archaea) into risk groups”) as cited by Nelkner et al. (2019).

**Additional file 5.** **Table S5**. List of antibiotic resistance genes (ARG) with query number of ≥ 16 harbored by known human microbial pathogens/colonizers that were detected in soil microbiomes of *Abutilon fruticosum*. N/A refers to ARGs that are not prevalent in known human bacterial pathogens/colonizers. These ARGs were not analyzed further as the risk of their direct transfer to human bacterial pathogens/colonizers is low. Species in blue are not in the list of human pathogens/colonizers, while that in red refers to a microbe acting as a biocontrol agent as described (Nelkner et al. 2019).

**Additional file 6.** **Table S6**. Query number of non-redundant antibiotic resistance genes (ARGs) of the different antimicrobial resistance mechanisms (AMR) at the phylum level of soil microbiomes of *Abutilon fruticosum*. Rows in red refer to less abundant mechanisms that were not analyzed further.

**Additional file 7.** **Table S7**. Abundance of non-redundant gene queries at bacterial genus/species level of rhizosphere (R) and bulk (S) soil microbiomes of *Abutilon fruticosum*.

**Additional file 8.** **Table S8**. Abundance of *Mycobacterium* genus/species in terms of non-redundant gene queries of rhizosphere (R) and bulk (S) soil microbiomes of *Abutilon fruticosum*. Bold rows refer to bacterial pathogens/colonizers whose ARGs are prevalent in human. The total list of human bacterial pathogens/colonizers is shown in Table S4.

**Additional file 9.** **Table S9**. Abundance of *Vibrio* genus/species in terms of non-redundant gene queries of rhizosphere (R) and bulk (S) soil microbiomes of *Abutilon fruticosum*. Bold row refers to a bacterial pathogen/colonizer whose ARGs are prevalent in human. The total list of human bacterial pathogens/colonizers is shown in Table S4.

**Additional file 10.** **Table S10**. Abundance of *Klebsiella* genus/species in terms of non-redundant gene queries of rhizosphere (R) and bulk (S) soil microbiomes of *Abutilon fruticosum*. Bold row refers to a bacterial pathogen/colonizer whose ARGs are prevalent in human. The total list of human bacterial pathogens/colonizers is shown in Table S4.

**Additional file 11.** **Table S11**. Abundance of *Stenotrophomonas* genus/species in terms of non-redundant gene queries of rhizosphere (R) and bulk (S) soil microbiomes of *Abutilon fruticosum*. Bold row refers to a bacterial pathogen/colonizer whose ARGs are prevalent in human. The total list of human bacterial pathogens/colonizers is shown in Table S4.

**Additional file 12.** **Table S12**. Abundance of *Pseudomonas* genus/species in terms of non-redundant gene queries of rhizosphere (R) and bulk (S) soil microbiomes of *Abutilon fruticosum*. Bold rows refer to bacterial pathogens/colonizers whose ARGs are prevalent in human. The total list of human bacterial pathogens/colonizers is shown in Table S4.

**Additional file 13.** **Table S13**. Abundance of *Nocardia* genus/species in terms of non-redundant gene queries of rhizosphere (R) and bulk (S) soil microbiomes of *Abutilon fruticosum*. Bold rows refer to bacterial pathogens/colonizers whose ARGs are prevalent in human. The total list of human bacterial pathogens/colonizers is shown in Table S4.

**Additional file 14.** **Table S14**. Abundance of *Salmonella* genus/species in terms of non-redundant gene queries of rhizosphere (R) and bulk (S) soil microbiomes of *Abutilon fruticosum*. Bold row refers to a bacterial pathogen/colonizer whose ARGs are prevalent in human. The total list of human bacterial pathogens/colonizers is shown in Table S4.

**Additional file 15.** **Table S15**. Abundance of *Escherichia* genus/species in terms of non-redundant gene queries of rhizosphere (R) and bulk (S) soil microbiomes of *Abutilon fruticosum*. Bold rows refer to bacterial pathogens/colonizers whose ARGs are prevalent in human. The total list of human bacterial pathogens/colonizers is shown in Table S4.

**Additional file 16.** **Table S16**. Abundance of *Citrobacter* genus/species in terms of non-redundant gene queries of rhizosphere (R) and bulk (S) soil microbiomes of *Abutilon fruticosum*. Bold rows refer to bacterial pathogens/colonizers whose ARGs are prevalent in human. The total list of human bacterial pathogens/colonizers is shown in Table S4.

**Additional file 17.** **Table S17**. Abundance of *Serratia* genus/species in terms of non-redundant gene queries of rhizosphere (R) and bulk (S) soil microbiomes of *Abutilon fruticosum*. Bold row refers to a bacterial pathogen/colonizer whose ARGs are prevalent in human. The total list of human bacterial pathogens/colonizers is shown in Table S4.

**Additional file 18.** **Table S18**. Abundance of *Shigella* genus/species in terms of non-redundant gene queries of rhizosphere (R) and bulk (S) soil microbiomes of *Abutilon fruticosum*. Bold rows refer to bacterial pathogens/colonizers whose ARGs are prevalent in human. The total list of human bacterial pathogens/colonizers is shown in Table S4.

**Additional file 19.** **Table S19**. Abundance of *Cronobacter* genus/species in terms of non-redundant gene queries of rhizosphere (R) and bulk (S) soil microbiomes of *Abutilon fruticosum*. Bold row refers to a bacterial pathogen/colonizer whose ARGs are prevalent in human. The total list of human bacterial pathogens/colonizers is shown in Table S4.

**Additional file 20.** **Table S20**. Abundance of *Bifidobacterium* genus/species in terms of non-redundant gene queries of rhizosphere (R) and bulk (S) soil microbiomes of *Abutilon fruticosum*. Bold row refers to a bacterial pathogen/colonizer whose ARGs are prevalent in human. The total list of human bacterial pathogens/colonizers is shown in Table S4.

**Additional file 21.** **Table S21**. Number of non-redundant gene queries of bacterial genera with species existing in the list of bacterial human pathogens/colonizers shown in Table S4 in rhizosphere (R) and bulk (S) soil microbiomes of *Abutilon fruticosum*.

**Additional file 22.** **Table S22**. Information retrieved from CARD site (https://card.mcmaster.ca/ontology/) for the top highly abundant ARGs (> 15 gene queries) in samples of rhizosphere and bulk soil microbiomes of *Abutilon fruticosum*.

**Additional file 23.** **Table S23**. Number of the most abundant non-redundant antibiotic resistance gene queries (ARGs) of rhizosphere (R) and bulk (S) soil microbiomes of *Abutilon fruticosum*. These ARGs are prevalent in bacterial human pathogens or colonizers.

**Additional file 24.** **Table S24**. Highly abundant non-redundant antibiotic resistance gene queries (ARGs) in rhizosphere (R) and bulk (S) soil microbiomes of *Abutilon fruticosum*. These ARGs are prevalent in bacterial human pathogens or colonizers.

## Data Availability

Additional files Data can be accessed at https://drive.google.com/drive/folders/1V-41wWnFGRJ6eWzcZXlARQSTwaMomMAv?usp=share_link
