# Correction: Abundant resistome determinants in rhizosphere soil of the wild plant *Abutilon fruticosum*

**DOI:** 10.1186/s13568-023-01606-y

**Published:** 2023-09-27

**Authors:** Wafa A. Alshehri, Aala A. Abulfaraj, Mashael D. Alqahtani, Maryam M. Alomran, Nahaa M. Alotaibi, Khairiah Alwutayd, Abeer S. Alouf, Fatimah M. Alshehrei, Khulood F. Alabbosh, Sahar A. Alshareef, Ruba A. Ashy, Mohammed Y. Refai, Rewaa S. Jalal

**Affiliations:** 1 Department of Biology, College of Science, University of Jeddah, 21493 Jed-Dah, Saudi Arabia; 2 Biological Sciences Department, College of Science & Arts, King Abdulaziz University, 21911 Rabigh, Saudi Arabia; 3 Department of Biology, College of Science, Princess Nourah bint Abdulrahman University, P.O.Box 84428, 11671 Riyadh, Saudi Arabia; 4 Department of Biology, Jumum College University, Umm Al-Qura University, P.O. Box 7388, 21955 Makkah, Saudi Arabia; 5 Department of Biology, College of Science, University of Hail, Hail, Saudi Arabia; 6 Department of Biology, College of Science and Arts at Khulis, University of Jeddah, 21921 Jeddah, Saudi Arabia; 7 Department of Biochemistry, College of Science, University of Jeddah, 21493 Jeddah, Saudi Arabia

**Correction: AMB Express (2023) 13:92** 10.1186/s13568-023-01597-w

Following publication of the original article (Alshehri et al. 2023), the author noticed an error in the funding number.

The Project number should be “PNURSP2023R356” instead of “PNURSP2023R355”. This has now been corrected in this erratum.

**Funding**

Princess Nourah bint Abdulrahman University Researchers Supporting Project number (PNURSP2023R356), Princess Nourah bint Abdulrahman University, Riyadh, Saudi Arabia.

The original article (Alshehri et al. 2023) has been corrected.
