# Supplementary material for: Abundant resistome determinants in rhizosphere soil of the wild plant Abutilon fruticosum
Source: AMB Express. 2023 Aug 30;13:92. doi: 10.1186/s13568-023-01597-w (PMC10469157; doi:10.1186/s13568-023-01597-w)
Supplement: Supplementary file 22 — Additional file 22. Table S22. Information retrieved from CARD site (https://card.mcmaster.ca/ontology/) for the top highly abundant ARGs (> 15 gene queries) in samples of rhizosphere and bulk soil microbiomes of Abutilon fruticosum. [file 13568_2023_1597_MOESM22_ESM.docx]

Table S22. Information retrieved from CARD site (<https://card.mcmaster.ca/ontology/>) for the top highly abundant ARGs (> 15 gene queries) in samples of rhizosphere and bulk soil microbiomes of *Abutilon fruticosum*.

| **CARD Short Name** | ***mtrA*** |
| --- | --- |
| Accession | ARO:3000816 |
| Definition | MtrA is a transcriptional activator of the MtrCDE multidrug efflux pump of Neisseria gonorrhoeae. |
| AMR Gene Family | [resistance-nodulation-cell division (RND) antibiotic efflux pump](https://card.mcmaster.ca/ontology/36005) |
| Drug Class | [penam](https://card.mcmaster.ca/ontology/36017), [macrolide antibiotic](https://card.mcmaster.ca/ontology/35919) |
| Resistance Mechanism | [antibiotic efflux](https://card.mcmaster.ca/ontology/36001) |
| Publications | Rouquette C, et al. (1999). Induction of the mtrCDE-encoded efflux pump system of *Neisseria gonorrhoeae* requires MtrA, an AraC-like protein. Mol Microbiol 33(3): 651-658. |
| **CARD Short Name** | ***soxR*** |
| Accession | ARO:3004107 |
| Definition | SoxR is a redox-sensitive transcriptional activator that induces expression of a small regulon that includes the RND efflux pump-encoding operon mexGHI-opmD. SoxR was shown to be activated by pyocyanin. |
| AMR Gene Family | [major facilitator superfamily (MFS) antibiotic efflux pump](https://card.mcmaster.ca/ontology/36003), [resistance-nodulation-cell division (RND) antibiotic efflux pump](https://card.mcmaster.ca/ontology/36005) |
| Drug Class | [tetracycline antibiotic](https://card.mcmaster.ca/ontology/36189), [fluoroquinolone antibiotic](https://card.mcmaster.ca/ontology/35920), [penam](https://card.mcmaster.ca/ontology/36017), [glycylcycline](https://card.mcmaster.ca/ontology/35960), [rifamycin antibiotic](https://card.mcmaster.ca/ontology/36296), [cephalosporin](https://card.mcmaster.ca/ontology/35951), [phenicol antibiotic](https://card.mcmaster.ca/ontology/36526), [disinfecting agents and antiseptics](https://card.mcmaster.ca/ontology/43746) |
| Resistance Mechanism | [antibiotic efflux](https://card.mcmaster.ca/ontology/36001) |
| Publications | Sakhtah H., et al. (2016). The *Pseudomonas aeruginosa* efflux pump MexGHI-OpmD transports a natural phenazine that controls gene expression and biofilm development. Proc. Natl. Acad. Sci. U.S.A., 113: E3538- E3547  Palma M., et al. (2005). *Pseudomonas aeruginosa* SoxR does not conform to the archetypal paradigm for SoxR-dependent regulation of the bacterial oxidative stress adaptive response. Infect Immun, 73: 2958-2966.  Dietrich L.E., et al. (2006). The phenazine pyocyanin is a terminal signaling factor in the quorum sensing network of *Pseudomonas aeruginosa*. Mol. Microbiol., 61: 1308-1321. |
| **CARD Short Name** | ***vanRO*** |
| Accession | ARO:3002930 |
| Definition | Also known as vanRO, is a vanR variant found in the vanO gene cluster. |
| AMR Gene Family | [glycopeptide resistance gene cluster](https://card.mcmaster.ca/ontology/36373), [vanR](https://card.mcmaster.ca/ontology/36713) |
| Drug Class | [glycopeptide antibiotic](https://card.mcmaster.ca/ontology/36220) |
| Resistance Mechanism | [antibiotic target alteration](https://card.mcmaster.ca/ontology/35997) |
| Publications | Gudeta DD, et al. 2014. vanO, a new glycopeptide resistance operon in environmental *Rhodococcus equi* isolates. Antimicrob Agents Chemother 58(3): 1768-1770. |
| **CARD Short Name** | ***golS*** |
| Accession | ARO:3000504 |
| Definition | GolS is a regulator activated by the presence of golD, and promotes the expression of the MdsABC efflux pump |
| AMR Gene Family | [resistance-nodulation-cell division (RND) antibiotic efflux pump](https://card.mcmaster.ca/ontology/36005) |
| Drug Class | [phenicolantibiotic](https://card.mcmaster.ca/ontology/36526), [monobactam](https://card.mcmaster.ca/ontology/35923), [penam](https://card.mcmaster.ca/ontology/36017), [cephalosporin](https://card.mcmaster.ca/ontology/35951), [cephamycin](https://card.mcmaster.ca/ontology/35962), [carbapenem](https://card.mcmaster.ca/ontology/35939) |
| Resistance Mechanism | [antibiotic efflux](https://card.mcmaster.ca/ontology/36001) |
| Publications | Pontel LB, et al. (2007). GolS controls the response to gold by the hierarchical induction of *Salmonella*-specific genes that include a CBA efflux-coding operon. Mol Microbiol 66(3): 814-825.  Perez Audero ME, et al. (2010). Target transcription binding sites differentiate two groups of MerR-monovalent metal ion sensors. Mol Microbiol 78(4): 853-865. |

| **CARD Short Name** | ***rbpA*** |
| --- | --- |
| Accession | ARO:3000245 |
| Definition | RNA-polymerase binding protein which confers resistance to rifampin. |
| AMR Gene Family | [RbpA bacterial RNA polymerase-binding protein](https://card.mcmaster.ca/ontology/41407) |
| Drug Class | [rifamycin antibiotic](https://card.mcmaster.ca/ontology/36296) |
| Resistance Mechanism | [antibiotic target protection](https://card.mcmaster.ca/ontology/35999) |
| Publications | Newell KV, et al. (2006). The RNA polymerase-binding protein RbpA confers basal levels of rifampicin resistance on *Streptomyces coelicolor*. Mol Microbiol 60(3): 687-696.  Dey A, et al. (2011). Molecular insights into the mechanism of phenotypic tolerance to rifampicin conferred on mycobacterial RNA polymerase by MsRbpA. Microbiology 157(PT 7): 2056-2071. |
| **CARD Short Name** | ***kdpE*** |
| Accession | ARO:3003841 |
| Definition | kdpE is a transcriptional activator that is part of the two-component system KdpD/KdpE that is studied for its regulatory role in potassium transport and has been identified as an adaptive regulator involved in the virulence and intracellular survival of pathogenic bacteria. kdpE regulates a range of virulence loci through direct promoter binding. |
| AMR Gene Family | [kdpDE](https://card.mcmaster.ca/ontology/41098) |
| Drug Class | [aminoglycoside antibiotic](https://card.mcmaster.ca/ontology/35935) |
| Resistance Mechanism | [antibiotic efflux](https://card.mcmaster.ca/ontology/36001) |
| Publications | Hirakawa H, et al. (2003). Comprehensive studies of drug resistance mediated by overexpression of response regulators of two-component signal transduction systems in *Escherichia coli*. J Bacteriol 185(6): 1851-1856.  Freeman ZN, et al. (2013). The KdpD/KdpE two-component system: integrating K⁺ homeostasis and virulence. PLoS Pathog. 9(3):e1003201. |
| **CARD Short Name** | ***rpoB2*** |
| Accession | ARO:3000501 |
| Definition | Due to gene duplication, the genomes of Nocardia species include both rifampin-sensitive beta-subunit of RNA polymerase (rpoB) and rifampin-resistant beta-subunit of RNA polymerase (rpoB2) genes, with ~88% similarity between the two gene products. Expression of the rpoB2 variant results in replacement of rifampin sensitivity with rifampin resistance. |
| AMR Gene Family | [rifamycin-resistant beta-subunit of RNA polymerase (rpoB)](https://card.mcmaster.ca/ontology/36349) |
| Drug Class | [rifamycin antibiotic](https://card.mcmaster.ca/ontology/36296) |
| Resistance Mechanism | [antibiotic target alteration](https://card.mcmaster.ca/ontology/35997) |
| Publications | Ishikawa J, et al. (2006). Contribution of rpoB2 RNA polymerase beta subunit gene to rifampin resistance in *Nocardia* species. Antimicrob Agents Chemother 50(4): 1342-1346. |
| **CARD Short Name** | ***arr-1*** |
| Accession | ARO:3002846 |
| Definition | arr-1 is a chromosome-encoded ribosyltransferase found in *Mycolicibacterium smegmatis* |
| AMR Gene Family | [rifampin ADP-ribosyltransferase (Arr)](https://card.mcmaster.ca/ontology/36529) |
| Drug Class | [rifamycin antibiotic](https://card.mcmaster.ca/ontology/36296) |
| Resistance Mechanism | [antibiotic inactivation](https://card.mcmaster.ca/ontology/36000) |
| Publications | Quan, S., et al. (1997). Ribosylative inactivation of rifampin by *Mycobacterium smegmatis* is a principal contributor to its low susceptibility to this antibiotic. Antimicrob Agents Chemother, 41: 2456-2460. |

| **CARD Short Name** | ***efrA*** |
| --- | --- |
| Accession | ARO:3003948 |
| Definition | efrA is a part of the EfrAB efflux pump, and both efrA and efrB are necessary to confer drug resistance |
| AMR Gene Family | [ATP-binding cassette (ABC) antibiotic efflux pum](https://card.mcmaster.ca/ontology/36002)p |
| Drug Class | [fluoroquinolone antibiotic](https://card.mcmaster.ca/ontology/35920), [macrolide antibiotic](https://card.mcmaster.ca/ontology/35919), [rifamycin antibioti](https://card.mcmaster.ca/ontology/36296)c |
| Resistance Mechanism | [antibiotic efflux](https://card.mcmaster.ca/ontology/36001) |
| Publications | Lavilla Lerma L, et al. (2014). Role of EfrAB efflux pump in biocide tolerance and antibiotic resistance of *Enterococcus faecalis* and *Enterococcus faecium* isolated from traditional fermented foods and the effect of EDTA as EfrAB inhibitor. Food Microbiol. 44:249-57.  Lee EW, et al. (2003). EfrAB, an ABC multidrug efflux pump in *Enterococcus faecalis*. Antimicrob Agents Chemother 47(12): 3733-3738. |
| **CARD Short Name** | ***Bifidobacterium bifidum ileS*** |
| Accession | ARO:3003730 |
| Definition | Bifidobacteria have an intrinsically resistant form of ileS (isoleucyl-tRNA synthetase) that confers resistance to mupirocin |
| AMR Gene Family | [antibiotic-resistant isoleucyl-tRNA synthetase (ileS)](https://card.mcmaster.ca/ontology/36585) |
| Drug Class | [mupirocin-like antibiotic](https://card.mcmaster.ca/ontology/45733) |
| Resistance Mechanism | [antibiotic target alteration](https://card.mcmaster.ca/ontology/35997) |
| Publications | Serafini F, et al. (2011). Insights into physiological and genetic mupirocin susceptibility in bifidobacteria. Appl Environ Microbiol 77(9): 3141-3146. |
